# Inflammatory Bowel Disease

**DOI:** 10.1093/emph/eou017

**Published:** 2014-04-18

**Authors:** Shona J. Lee, Rick M. Maizels

**Affiliations:** ^1^Centre for African Studies and ^2^Institute of Immunology and Infection Research, University of Edinburgh

## Inflammatory bowel disease

**Figure 1.**
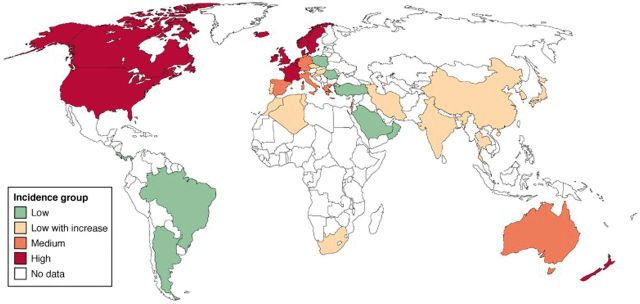
Global incidence of IBD. Reprinted with permission from Elsevier [2].

Inflammatory bowel disease (IBD) is an immunological disorder, encompassing Crohn’s disease and ulcerative colitis, which are characterized by chronic intestinal inflammation targeted at harmless commensal bacteria and food antigens. Although the aetiology of IBD remains unclear, environmental factors in susceptible individuals appear to trigger immunological responses that inflame and damage tissues of the digestive tract. Prevalence of IBD is markedly higher in industrialized and affluent countries [1] (see Fig. 1). Evidence of a major underlying role for genetic predisposition to IBD raises the likelihood that the origins of disease and the susceptibility of the current human ‘immunome’ is the evolutionary consequence of marked and prolonged genetic selective pressure exerted by infectious pathogens [3].

## Evolutionary perspectives

The Hygiene Hypothesis suggests that increasing allergic and autoimmune diseases are related to improved hygiene, e.g. the absence of helminth infections in more developed countries [1]. Notably, allergic diseases are less common where parasites are rife, while the elimination of helminths can increase the atopic response to allergens. Our human ancestors would have been exposed to such infections causing, over evolutionary time, the human immune system to be optimally calibrated for their presence [3].

In helminth-free settings, our immune system may be maladapted and, where free of the dampening effect of helminths, may over-react to harmless antigens. Multiple studies have revealed that helminths suppress a broad range of inflammatory responses [4], which may explain why exposure to helminths such as *Trichuris suis* (whipworm) has been reported to be well tolerated by patients and improve IBD without any overt side-effects in an open label trial [5, 6]. These therapies are now being evaluated in double-blind clinical trials.

## Future clinical implications

As the precise aetiology of IBD is unknown, no curative treatment is currently available [2]. If positive results from pilot trials are confirmed in larger efficacy trials, then it is feasible that helminths, applied in a controllable clinical setting, could relieve inflammatory disease. The immunoregulatory effects of microbial exposure have moved beyond hypothesis, with focus now turning to their specific mechanisms [7], and a greater understanding of the immunological effects of helminths**.** Ultimately, this field of research may lead to therapeutic treatments for patients suffering from inflammatory, autoimmune or allergic diseases [8].

## References

[1] Economou M, Pappas G (2008). New global map of Crohn's disease: Genetic, environmental, and socioeconomic correlations. Inflamm Bowel Dis.

[2] Cosnes J, Gower-Rousseau C, Seksik P (2011). Epidemiology and natural history of inflammatory bowel diseases. Gastroenterology.

[3] Maizels RM (2005). Infections and allergy—helminths, hygiene and host immune regulation. Curr Opin Immunol.

[4] McSorley HJ, Maizels RM (2012). Helminth infections and host immune regulation. Clin Microbiol Rev.

[5] Summers RW, Elliott DE, Qadir K (2003). *Trichuris suis* seems to be safe and possibly effective in the treatment of inflammatory bowel disease. Am J Gastroenterol.

[6] Summers RW, Elliott DE, Urban JF (2005). *Trichuris suis* therapy in Crohn's disease. Gut.

[7] Rook GAW, Raison CL, Lowry CA (2014). Microbial ‘Old Friends’, immunoregulation and socio-economic status. Clin and Exp Immunol.

[8] Weinstock JV, Elliott DE (2013). Translatability of helminth therapy in inflammatory bowel diseases. Int J Parasitol.

